# ARCTIC-T: Annular Reduction by Cinching With Transcatheter Tricuspid Edge-to-Edge Repair to Facilitate Concomitant Transcatheter Tricuspid Valve Replacement

**DOI:** 10.1016/j.shj.2026.101055

**Published:** 2026-06-05

**Authors:** Dennis Mehrkens, Maria Isabel Körber, Jonathan Curio, Sebastian Röschl, Stephan Baldus, Roman Pfister, Matti Adam

**Affiliations:** a Department of Cardiology, Heart Center Cologne, University of Cologne, Faculty of Medicine and University Hospital, Cologne, Germany; b Klinikum Leverkusen, Clinic of Cardiology, Leverkusen, Germany

**Keywords:** Annuloplasty, Combined procedure, Transcatheter tricuspid edge-to-edge repair (T-TEER), Transcatheter tricuspid valve replacement (TTVR)

## Abstract

• This report illustrates an approach using transcatheter tricuspid edge-to-edge repair–mediated annular remodeling to facilitate transcatheter tricuspid valve replacement.
• Commissural leaflet approximation may improve annular geometry and device anchoring.
• The annular reduction by cinching with transcatheter tricuspid edge-to-edge repair to facilitate concomitant transcatheter tricuspid valve replacement strategy represents a first-in-human, hypothesis-generating concept.

Transcatheter therapies, including transcatheter tricuspid valve replacement (TTVR), are increasingly used. However, excessive annular dilatation may limit feasibility. Targeted annular remodeling using transcatheter tricuspid edge-to-edge repair (T-TEER) may facilitate valve implantation. We describe the annular reduction by cinching with transcatheter edge-to-edge repair in the tricuspid position (ARCTIC-T) strategy, in which intentional annular cinching enables concomitant TTVR within the same procedure.^1^^,^^2^

Despite receiving optimal medical therapy, an 82-year-old male presented with symptomatic severe functional tricuspid regurgitation (TR) (New York Heart Association class III) with recurrent right-sided heart failure hospitalizations. A prior T-TEER procedure at an external center was aborted due to insufficient TR reduction. Computed tomography at that time demonstrated borderline annular dimensions (annular diameter 53.7 mm) without relevant right ventricular enlargement (right ventricular diameter 63.6 mm), suggesting initial eligibility for TTVR.

Four months later, despite optimized medical therapy, intraprocedural three-dimensional transesophageal echocardiography at our institution revealed torrential functional TR with progressive annular dilatation (septal-lateral 57 mm, anteroposterior 51.7 mm, perimeter 173 mm), raising concerns regarding stable device anchoring for isolated TTVR.

A stepwise strategy was pursued with parallel implantation of two PASCAL ACE devices in the anteroseptal commissure, achieving leaflet approximation and annular cinching (Figure 1, Video 1) to enhance anchoring stability. Reassessment demonstrated a reduction in septal-lateral diameter from 57 mm to 46 mm (–19.3%) and annular perimeter from 173 mm to 159 mm (–8%), whereas the anteroposterior diameter remained unchanged at 51.9 mm. Despite modest TR reduction, annular cinching improved geometry for TTVR and allowed downsizing from 56 to 52 mm. Consequently, a 52-mm EVOQUE prosthesis was implanted. The previously placed T-TEER devices contributed to annular remodeling and provided additional stabilization during deployment. Discharge echocardiography demonstrated complete elimination of TR without relevant paravalvular leakage and low transvalvular gradients (1.6 mmHg).

**Figure 1:**
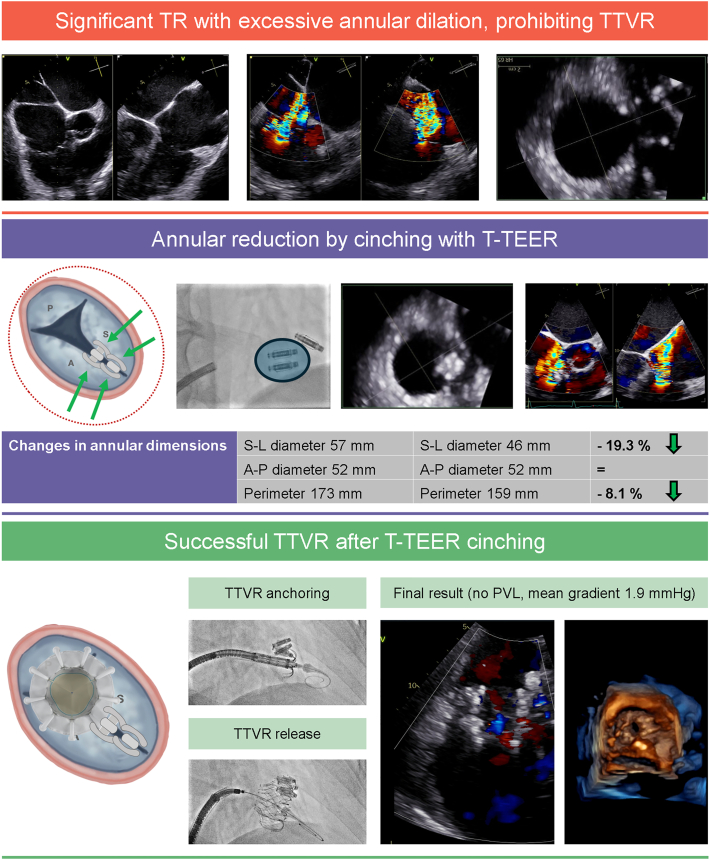
**ARCTIC-T: Annular reduction by cinching with transcatheter edge-to-edge repair in the tricuspid position, enabling subsequent transcatheter tricuspid valve replacement.** Abbreviations: A-P, anteroposterior; PVL, paravalvular leakage; S-L, septal-lateral; TR, tricuspid regurgitation; T-TEER, tricuspid transcatheter edge-to-edge repair; TTVR, transcatheter tricuspid valve replacement.

The strategy was planned with computed tomography and guided by intraprocedural imaging. The anteroseptal commissure was selected according to the zipping technique, to reduce septal-lateral dimensions, which was relevant for EVOQUE anchoring in this case. Intraprocedurally, no strict numerical target for annular reduction was predefined. Potential risks included leaflet injury, single leaflet device attachment, and device interaction, which were mitigated by careful imaging guidance and the use of 2 T-TEER devices.

This report describes a single, highly selected case with acute procedural success. No conclusions regarding reproducibility, safety, or broader applicability can be drawn.

This first-in-human concomitant application of T-TEER and TTVR demonstrates that intentional annular remodeling may enable definitive valve replacement within a single procedure.

## Funding

The authors have no funding to report.

## Disclosure Statement

Dennis Mehrkens reports lecture fees and travel support by 10.13039/100006520Edwards Lifesciences and 10.13039/100000042Amgen. Maria Isabel Körber has received travel support and lecture fees from 10.13039/100019998JenaValve, 10.13039/100006520Edwards Lifesciences, and 10.13039/100000046Abbott. Roman Pfister reports lecture fees by 10.13039/100006520Edwards Lifesciences and Abbot Vascular and consultant fees by 10.13039/100006520Edwards Lifesciences. Jonathan Curio has received research grant support from 10.13039/100008497Boston Scientific; has served on an advisory board for 10.13039/100004374Medtronic; and has received travel support from Laralab and Cardiovalve. Stephan Baldus reports lecture fees from JenaValve and lecture and speaker fees from 10.13039/100006520Edwards Lifesciences. Matti Adam reports consulting fees from 10.13039/100000046Abbott, JenaValve Technology, 10.13039/100006520Edwards Lifesciences, Haemonetics, 10.13039/100004374Medtronic, and Meril.

The other authors had no conflicts to declare.

## Supplementary material

Video 1 Baseline imaging followed by annular reduction by cinching with transcatheter edge-to-edge repair in the tricuspid position (ARCTIC-T), enabling subsequent transcatheter tricuspid valve replacement.
